# Comparison of Auto Sampling and Passive Sampling Methods for SARS-CoV-2 Detection in Wastewater

**DOI:** 10.3390/pathogens11030359

**Published:** 2022-03-16

**Authors:** Melissa Wilson, Yuanyuan Qiu, Jiaao Yu, Bonita E. Lee, David T. McCarthy, Xiaoli Pang

**Affiliations:** 1 Department of Laboratory Medicine and Pathology, University of Alberta, Edmonton, AB T6G 2B7, Canada; mmwilson@ualberta.ca (M.W.); jiaao3@ualberta.ca (J.Y.); xiao-li.pang@albertaprecisionlabs.ca (X.P.); 2 Department of Pediatrics, University of Alberta, Edmonton, AB T6G 1C9, Canada; bonita.lee@albertahealthservices.ca; 3 Environmental and Public Health Microbiology Laboratory (EPHM Lab), Department of Civil Engineering, Monash University, Clayton, VIC 3800, Australia; david.mccarthy@monash.edu; 4 Public Health Laboratories (ProvLab), Alberta Precision Laboratories (APL), Edmonton, AB T6G 2J2, Canada

**Keywords:** SARS-CoV-2, wastewater, passive sampler, autosampler, RT-qPCR

## Abstract

Wastewater-based surveillance is emerging as an important tool for the COVID-19 pandemic trending. Current methods of wastewater collection, such as grab and auto-composite sampling, have drawbacks that impede effective surveillance, especially from small catchments with limited accessibility. Passive samplers, which are more cost-effective and require fewer resources to process, are promising candidates for monitoring wastewater for SARS-CoV-2. Here, we compared traditional auto sampling with passive sampling for SARS-CoV-2 detection in wastewater. A torpedo-style 3D-printed passive sampler device containing both cotton swabs and electronegative filter membranes was used. Between April and June 2021, fifteen passive samplers were placed at a local hospital’s wastewater outflow alongside an autosampler. Reverse transcription and quantitative polymerase chain reaction (RT-qPCR) was used to detect SARS-CoV-2 in the samples after processing and RNA extraction. The swab and membrane of the passive sampler showed similar detection rates and cycle threshold (Ct) values for SARS-CoV-2 RNA for the N1 and N2 gene targets. The passive method performed as well as the grab/auto sampling, with no significant differences between N1 and N2 Ct values. There were discrepant results on two days with negative grab/auto samples and positive passive samples, which might be related to the longer duration of passive sampling in the study. Overall, the passive sampler was rapid, reliable, and cost-effective, and could be used as an alternative sampling method for the detection of SARS-CoV-2 in wastewater.

Wastewater-based surveillance (WBS) of SARS-CoV-2 has become a promising tool to monitor the prevalence of COVID-19 in the community because infected persons shed SARS-CoV-2 in feces [1,2,3,4,5,6], leading to the presence of SARS-CoV-2 virus and RNA in wastewater [7,8,9,10]. Currently, many studies focus on wastewater collected from wastewater treatment plants (WWTP). However, studies focusing on small catchments, such as long-term care facilities, schools, hospitals, apartment complexes, etc., are important for timely monitoring and public health actions to the targeted population. These smaller catchments, particularly in remote communities, can present a challenge for wastewater sample collection.

Grab and 24 h composite automatic sampling are the two most common wastewater sampling methods. Grab sampling is convenient and easy, and can be collected from the outflow tracks of most catchments because installation of special equipment is not required. However, as the contents of wastewater vary considerably depending on the time of day and associated human activities, grab sampling may miss viral shedding discharges to sewers and provide less representative surveillance. To overcome this limitation, automatic samplers are installed to collect 24 h composite samples. Although composite samples catch variation of the wastewater over time, it is not always possible to use the automatic sampling method for small catchments due to several reasons, such as the high cost of equipment and maintenance, requirement for skilled personnel to install and power to operate the sampler, limited space for installation, difficulty to access the sampling site, challenges in areas with sub-zero temperatures, and low wastewater flow. Therefore, alternative sampling methods for small catchments may offer practical advantages.

Passive sampling methods, which often take the form of a modified “Moore swab” [11], combine the advantages of both grab and automatic sampling. A passive sampler is an abiotic device that may contain absorbent materials or membranes. It is placed in a targeted sewage catchment to capture viruses for a defined period of time. There are several benefits of using passive samplers. They are easy to deploy and collect from small catchments and do not require power to operate, and thus can be used in any accessible sewage line. Furthermore, passive samplers collect viruses over the entire stay time, so shedding events are unlikely to be missed. Passive samplers have been used to monitor wastewater for SARS-CoV-2 at an institutional level. Corchis-Schott et al., used a tampon as a Moore swab to monitor for COVID-19 cases at a university residence hall [12]. Upon receiving a positive sample, resident COVID-19 testing was initiated and contact tracing was used to prevent the spread of disease in the residence. Liu et al. compared Moore swabs and grab samples from a university hospital sewage line and found that passive sampling was more sensitive than grab sampling for SARS-CoV-2 detection [13]. Likewise, Rafiee et al., found that Moore swab passive sampling performed similarly to automatic composite sampling, and outperformed grab sampling, in terms of SARS-CoV-2 detection in wastewater from manholes of small communities [14].

Although Moore swab-style passive samplers have yielded promising results for COVID-19 surveillance, these passive samples are prone to disruption of the contact required between the swab and wastewater caused by solids in the wastewater [15]. Furthermore, Moore swabs may be lost or destroyed if impacted by solids flowing in the sewer line [16]. Researchers at Monash University in Melbourne developed a passive sampling device, which is composed of a 3D-printed torpedo-shaped shell housing two types of materials: absorbent cotton and negatively charged membranes [15]. This sampler is designed to sit in the sewer line and allow flow of wastewater through the housed materials. The passive sampling device was shown to be effective, even detecting SARS-CoV-2 in wastewater when the case rate of COVID-19 was low in the population [15]. Another passive sampling device called the “COVID-19 sewer cage” (COSCa) was recently developed to detect SARS-CoV-2 in wastewater [16]. Although studies showed the potential of using a passive sampler for virus detection in wastewater, there is limited information about their efficacy in the detection of SARS-CoV-2 in wastewater.

Our laboratory is currently conducting a large-scale wastewater monitoring project for COVID-19 that includes auto sampling at multiple wastewater treatment plants and smaller catchments [17]. Due to the disadvantages of auto sampling, we are interested in determining whether passive sampling poses a viable option for detecting SARS-CoV-2 in small catchments. In this study, we compare the performance of two types of materials in a passive sampling device and an automatic composite sampling method for SARS-CoV-2 detection in a small wastewater catchment at a local hospital.

As a proof-of-concept, a small bench-scale experiment was conducted to determine the efficiency of viral detection by two absorbent materials: gauze housed in a 3D-printed casing with holes to allow the flow-through of wastewater and feminine hygiene products (Tampax Pearl, Super). Two composite wastewater samples collected from a WWTP in Alberta were used. The absorbent materials were placed in a beaker containing 150 mL of the wastewater for 24 h at room temperature, with or without stirring to simulate water movement in an outflow. After 24 h, the tampons and gauze were removed from the wastewater and placed in the barrel of a 50 mL syringe. The syringe was plunged to remove as much liquid as possible from the absorbent materials into a 50 mL tube. The materials were further rinsed with PBS to obtain a final volume of 50 mL.

The samples were concentrated using Centricon^®^ Plus-70 centrifugal ultrafilters (30-kDa MWCO, Millipore, Burlington, MA, USA) [17]. Briefly, the pH of the samples was adjusted to 9.6–10.0, then vortexed for 30 s to release solid-bound virus and RNA into solution, followed by centrifugation at 4500× *g* for 10 min. After removing the solids, the supernatant pH was adjusted to neutral (6.9–7.4), then added into the Centricon^®^ ultrafilter cup and centrifuged at 3000× *g* to a final concentrate volume of 1 mL as previously described [18]. Alongside the passive sampling experiment, 100 mL of the wastewater samples were also processed using the same concentration method to determine the viral load in the samples.

RNA was extracted from 400 μL of the concentrated wastewater and passive samples using the MagMax^TM^ 96 viral isolation kit with the Kingfisher Flex automatic system to get a final elution volume of 100 μL. One-step reverse-transcription quantitative PCR (RT-qPCR) targeting two regions of the nucleocapsid protein gene of SARS-CoV-2 (N1 and N2), as well as the pepper mild mottle virus (PMMoV), a fecal indicator, were performed as previously described [17,19]. Each sample was tested for N1 and N2 genes in duplicate. A SARS-CoV-2 positive result was defined as a sample with two or more positive results out of the four PCR runs.

Both passive sampling materials were sufficient to detect SARS-CoV-2 (Table 1). The tampon samples yielded average N1 and N2 cycle threshold (Ct) values of 29.47 and 29.50 (sample 1, no stirring) and 28.81 and 29.68 (sample 2, no stirring). The addition of stirring did not affect viral detection, with similar average N1 and N2 Ct values observed for both samples (Table 1). The gauze material showed a higher Ct value compared to the tampon with average N1 and N2 Ct values of 31.15 and 31.15 (sample 1, no stirring) and 31.02 and 31.19 (sample 2, no stirring). This may be due to the lower adsorption capacity of gauze, based on the higher Ct value of PMMoV observed for the gauze compared to the tampon. Overall, the tampon showed better performance compared to the gauze for the detection of SARS-CoV-2 in wastewater, which was comparable to the direct detection from wastewater samples (Table 1). This proof-of-concept experiment confirmed the feasibility of passive sampling for monitoring SARS-CoV-2 in wastewater and prompted us to further evaluate the performance of the torpedo passive sampling device in a sewer catchment.

The torpedo passive sampler was provided by Dr. McCarthy, Monash University, Melbourne, Australia. Wastewater samples were collected twice a week from the manhole of a hospital located in the City of Edmonton, Canada from April to June 2021. A traditional automatic GLS sampler (Avensys Inc., Calgary, AB, Canada) was installed at the site to collect 24 h composite samples. With our study design of twice a week visit by study personnel at the manhole, passive samplers were deployed in the sewer at the time of wastewater sample collection, with the deployment time ranging from 48 to 144 h depending on the day of the week the sampler was placed into the manhole. Each passive sampling event was paired with the collection of a 24 h composite sample, where sampling started one day before collection and overlapped the last 24 h of the passive sampling period. If composite samples were not successfully collected because of weather or shallow wastewater flow, grab samples were taken. Once collected, samples including wastewater and the passive sampler (inside a Zip bag) were transported to the laboratory on ice and processed on the same day. A total of fifteen samples were collected using the passive sampler, along with eleven composite samples and four grab samples during the study period. Each passive sampler contained three cotton swabs and three electronegative filter membranes. One swab and one membrane were processed from each passive sampler, and the remaining materials were stored at −70 °C for future use. The swab or membrane was placed in a 2 mL tube with 502 μL Lysis/Binding Solution (MagMAX Viral RNA Isolation Kit, ThermoFisher, ON, Canada) and vortexed vigorously for two minutes. They were then removed from the tube, while sterile tweezers were used to squeeze the material against the inner wall of the tube to extract as much liquid as possible. The entire volume of the sample was used for nucleic acid extraction as described above. Grab or 24 h composite wastewater samples collected from the same site were processed in paralle with the Centricon^®^ concentration and nucleic acid extraction as described above. SARS-CoV-2 N1 and N2 genes, as well as PMMoV, were assessed by one-step RT-qPCR [19].

Among the 15 collected passive samples and grab/composite samples, SARS-CoV-2 was detected in 12 (80%) passive samples and 10 (67%) grab/composite samples (Table 2). The Ct values of grab/composite samples ranged from 26.6 to 32.1 for N1 (median Ct: 30.2) and 26.7 to 32.2 for N2 (median Ct: 30.5). For passive samples, the Ct values for the membrane ranged from 29.5 to 36.2 for N1 (median Ct: 31.6) and 30 to 35.9 for N2 (median Ct: 32.5), and ranged from 29.1 to 35.3 for N1 (median Ct: 32.9) and 30.4 to 36.4 for N2 (median Ct: 33.2) for the cotton swab (Table 2). There is no significant difference in the Ct value between grab/composite and passive samples, as well as membrane and cotton swab for SARS-CoV-2 N1 and N2 genes (Figure 1 and Figure 2) (*p* > 0.05, Wilcoxon signed-rank test). On those ten collection days where grab/composite samples showed detection of SARS-CoV-2, at least one passive sampler material collected on the same day also tested positive for SARS-CoV-2 (Table 3). There were two days where the grab/composite samples failed to detect SARS-CoV-2, while the passive samples were positive for SARS-CoV-2 (Table 3). Due to the study design, the passive samplers were placed in the sewage outflow for longer than 24 h, and this may have allowed the detection of viral shedding events prior to the start of the automatic sampling period. Additionally, the passive samplers are in continuous contact with the wastewater outflow and may catch shedding events between automatic sampling pulses.

PMMoV was detected in all the samples collected by both methods, indicating that fecal material was present in all samples. The Ct values of PMMoV obtained from the auto/grab samples (median Ct: 21.1) were significantly lower than the two passive sampling materials (combined median Ct: 25.5) (*p* < 0.05, Wilcoxon signed-rank test). Additionally, the swab had a significantly lower Ct value for PMMoV (median Ct: 25.0) than the membrane (median Ct: 26.0) (*p* < 0.05, Wilcoxon signed-rank test) (Table 2, Figure 3).

In our study, the cotton swab and electronegative membrane in the passive sampler had similar detection rates and Ct values for the two gene targets of SARS-CoV-2 (Table 2). However, using the same torpedo passive sampling devices, Schang et al., found that the electronegative membrane detected SARS-CoV-2 RNA more often than the cotton swab (41 and 25% of total samples were found to be positive, respectively) [15]. Several materials have been studied for SARS-CoV-2 detection using passive samplers. Modified Moore swabs constructed from cotton gauze or feminine hygiene products have been shown effective for viral detection [12,13]. Materials used in 3D-printed passive sampling devices include cotton swabs, medical gauze, cheesecloth, cellulose sponges, and electronegative membranes, with cheesecloth and electronegative membranes showing the best results in the COSCa devices [15,16].

Another factor to be considered is the various methodologies used to extract viral RNA from the absorbent materials in different studies. In our study, the Lysis/Binding Solution provided with the MagMAX Viral RNA Isolation Kit was used as this allowed us to directly add the solution to the RNA extraction assay alongside concentrated grab/composite wastewater samples. However, Hayes et al. experimented with different elution mixtures and concluded that a Tween^®^20-based buffer had the best performance [16]. For their tampon-style passive sampler, Corchis-Schott et al. did not use an elution buffer to extract viral particles and RNA from the absorbent material; rather, they used manual pressure with a 50 mL syringe to squeeze the existing liquid from the material [12]. Likewise, Liu et al. used manual pressure to squeeze liquid from the Moore swab but subsequently used a Tween-based buffer solution to remove the remaining particles from the swab [13]. A bead-beating step, as used by Schang et al., or other mechanical manipulation, may facilitate the removal of viral particles and RNA from the absorbent materials [15].

A limitation of passive samplers is the difficulty in quantifying the SARS-CoV-2 level in wastewater since the total volume of wastewater flowing through the passive sampler is unknown. To estimate the concentration of the viral genome in wastewater, the Ct value of SARS-CoV-2 target genes can be normalized to the fecal indicator, PMMoV. Corchis-Schott et al., calculated SARS-CoV-2 concentration in wastewater based on the ratio of SARS-CoV-2:PMMoV in passive samples, along with estimates of flow rate, wet fecal mass, and the number of infected people contributing to the outflow [12]. However, these metrics are difficult to estimate accurately, as flow rates vary with levels of activity throughout the day, especially in the drainage systems from smaller facilities [12]. Further, the number of infected individuals may not be known in community settings or during outbreaks.

Another important consideration is the deployment time of the passive sampler which varied from 48 h to 144 h in our study. Although the passive samplers were deployed in the wastewater flow for longer times compared to the autosampler, the similar Ct values of SARS-CoV-2 detected by the passive samplers suggest that increasing sampling time may not enhance total detection. This might be due to the dilution effect from a continuous flow of wastewater with varying levels of SARS-CoV-2 through the passive sampler, or limited adsorption capacity of the materials. On the other hand, the two discrepant positive passive samples and negative grab/auto samples (Table 3) suggest that a longer duration of sampling increased sensitivity to detect SARS-CoV-2. This trend was seen in a study by Liu et al., where Moore swab passive sampling had a higher SARS-CoV-2 detection rate than grab sampling, and passive sampling was sufficient to detect one to two COVID-19 cases in a residence building, perhaps due to an increased sampling time of up to 72 h [13]. Therefore, the deployment time ranges from 24–72 h would be ideal for the passive sampler.

The application of the passive sampling approach may depend on collection sites and wastewater parameters, such as water flow rate, solid content, etc. A sewer line with high water flow may cause the loss or damage of the passive sampler. Large amounts of solids in the wastewater may reduce the virus recovery efficiency due to inhibition in RNA extraction and the RT-qPCR process [16]. Therefore, the passive sampling method is more suitable for small sewage systems with low water flow, especially the manholes for specific facilities, such as schools, hospitals, university campuses, and long-term care facilities. The WBS of SARS-CoV-2 at smaller catchments allows for rapid public health reaction and may prevent disease transmission at an early stage.

Compared to traditional grab or automatic sampling methods, the passive sampler is more cost-effective, less labor-intensive, and has a shorter sample processing time before testing, in part because the costly and time-consuming step of sample concentration was not required for passive sampler processing. Requiring fewer resources makes passive sampling a good choice for long-term monitoring efforts and monitoring in resource-poor countries. Although the sample size is small in this study, our results demonstrate that the passive sampler has valuable potential for monitoring COVID-19 prevalence in small catchments. We recommend that passive sampling could be used as an alternative sampling method for the detection of SARS-CoV-2 in wastewater.

## Figures and Tables

**Figure 1:**
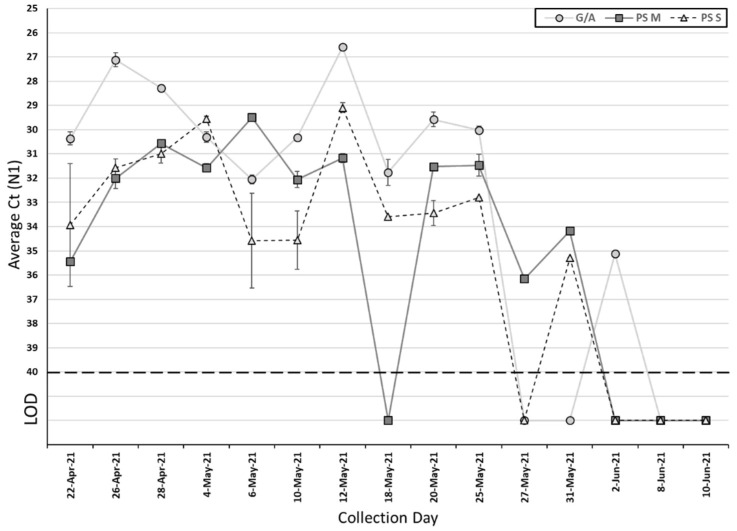
Average Ct for the SARS-CoV-2 N1 target using passive samplers and grab/autosamplers. G/A, grab/autosampler; PS M, passive sampler (membrane); PS S, passive sampler (swab); LOD, limit of detection. Error bar represents standard deviation.

**Figure 2:**
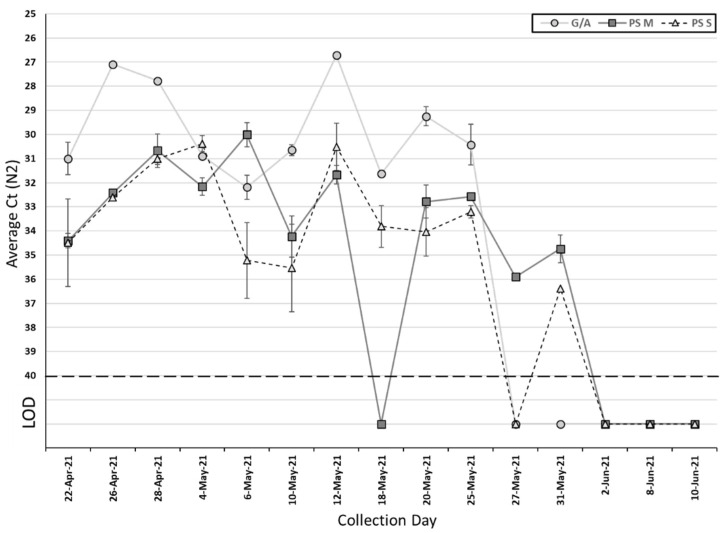
Average Ct for the SARS-CoV-2 N2 target using passive samplers and grab/autosamplers. G/A, grab/autosampler; PS M, passive sampler (membrane); PS S, passive sampler (swab); LOD, limit of detection. Error bar represents standard deviation.

**Figure 3:**
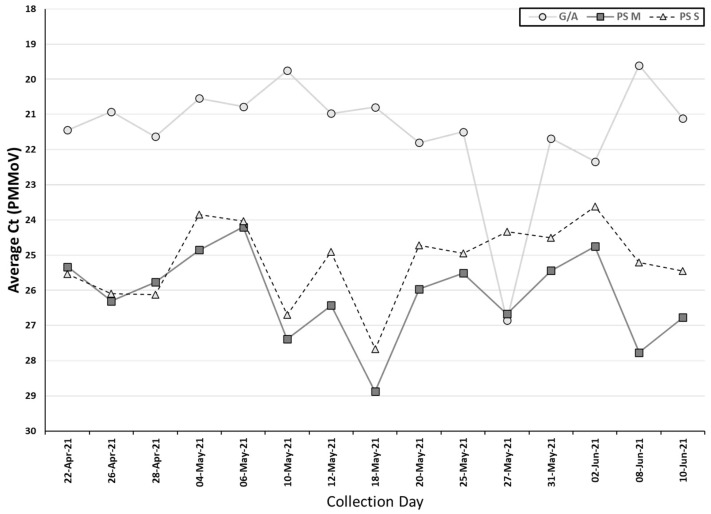
Ct value for the PMMoV using passive samplers and grab/autosamplers. G/A, grab/autosampler; PS M, passive sampler (membrane); PS S, passive sampler (swab).

**Table 1:** Comparison of the detection of SARS-CoV-2 and PMMoV by different sampling methods in the bench-scale study.

	Sample Type	N1 Ct, Mean	N2 Ct, Mean	PMMoV, Ct
Sample 1	100 mL wastewater	28.05	28.5	19.47
	Tampon, no stirring	29.47	29.5	20.83
	Tampon, stirring	29.91	29.29	20.63
	Gauze, no stirring	31.15	31.15	21.54
	Gauze, stirring	31.5	31.35	21.79
Sample 2	100 mL wastewater	27.2	27.32	19.41
	Tampon, no stirring	29.81	29.68	20.79
	Tampon, stirring	29.18	28.75	20.74
	Gauze, no stirring	31.02	31.19	22
	Gauze, stirring	30.93	30.26	21.72

PMMoV: Pepper mild mottle virus.

**Table 2:** SARS-CoV-2 detection in wastewater using passive samplers and grab/autosampler.

	Auto Sampler	Passive Sampler		
		Membrane	Swab	Membrane + Swab
Detection rate	10/15	11/15	11/15	12/15
N1 Ct, median	30.2	31.6	32.9	31.8
N1 Ct, range	26.5–35.1	29.5–36.2	29–36	29–36.2
N2 Ct, median	30.5	32.5	33.2	32.8
N2 Ct, range	26.7–32.6	29.7–35.9	29.8–36.8	29.7–36.8
PMMoV Ct, median	21.1	26	25	25.5
PMMoV Ct, range	19.2–26.9	24.2–28.9	23.6–27.7	23.6–28.9

**Table 3:** Detection of SARS-CoV-2 on each sampling day using passive samplers and grab/autosamplers. A positive result was determined when at least two out of four RT-qPCR tests for N1 and N2 targets were observed as positive. The symbol “+” represents the detection of SARS-CoV-2 in the sample; symbol “−” represents no detection of SARS-CoV-2.

Sampling Day	Auto Sampler (100 mL Wastewater)		Passive Sampler		
	SARS-CoV-2 Detection	Sample Type	SARS-CoV-2 Detection		Deployment Time (Hours)
			Membrane	Swab	
22 April 2021	+	Composite	+	+	48
26 April 2021	+	Composite	+	+	96
28 April 2021	+	Composite	+	+	48
4 May 2021	+	Composite	+	+	144
6 May 2021	+	Composite	+	+	48
10 May 2021	+	Composite	+	+	96
12 May 2021	+	Composite	+	+	48
18 May 2021	+	Composite	−	+	144
20 May 2021	+	Composite	+	+	48
25 May 2021	+	Composite	+	+	120
27 May 2021	−	Composite	+	−	48
31 May 2021	−	Grab	+	+	96
2 June 2021	−	Grab	−	−	48
8 June 2021	−	Grab	−	−	96
10 June 2021	−	Grab	−	−	48

## Data Availability

The data supporting the findings of this study are available within this article.
